# Demonstration of a Fizeau Directly-Imaging Sparse-Aperture Telescope with Pointing and Tracking Capabilities

**DOI:** 10.3390/mi14030569

**Published:** 2023-02-27

**Authors:** Liangzhu Yuan, Jianliang Shi, Yongmei Huang, Jinying Li, Piao Wen, Haotong Ma, Yang Li, Huayang Xia, Qiong Tu, Rongqi Ma

**Affiliations:** 1 Key Laboratory of Optical Engineering, Chinese Academy of Sciences, Chengdu 610200, China; 2 Institute of Optics and Electronics, Chinese Academy of Sciences, Chengdu 610200, China; 3 University of Chinese Academy of Sciences, Beijing 100049, China; 4 School of Electronic, Electrical and Communication Engineering, University of Chinese Academy of Sciences, Beijing 100049, China

**Keywords:** sparse-aperture telescope, Risley prism, pointing, tracking

## Abstract

At present, the majority of sparse-aperture telescopes (SATs) are unable to observe moving targets. In this paper, we describe the construction of and present the results obtained using a Fizeau directly-imaging sparse-aperture telescope (FDISAT) that permits pointing and the tracking of moving targets. The telescope comprises three sub-apertures, each of which is equipped with a Risley prism system that permits a maximum tracking range of 5° and has independent boresight adjustment capability. On targets in various positions, experiments with pointing and tracking are conducted. The maximum root-mean-square error (RMSE) of pointing in the sub-apertures was found to be 8.22 arcsec. When considering a target moving at 0.01°/s for approximately 320 s, the maximum RMSE of tracking in the sub-apertures was found to be 4.23 arcsec. The images obtained from the focal plane detector exhibit clear interference fringes while tracking. The experimental results demonstrate that the system can effectively track moving targets, providing a method for SAT observation of moving targets.

## 1. Introduction

The Rayleigh criterion indicates that the resolution of a telescope increases with its aperture size. However, there are practical difficulties in increasing the aperture size of a telescope imposed by both cost and manufacturing restrictions. SATs have been developed to overcome these restrictions.

In 1985, the Multiple Mirror Telescope, which comprises six sub-apertures with 1.8-m, was constructed by the University of Arizona [1]. In 1988, three sub-aperture telescopes with a combined aperture of 10 cm were created by the Air Force Weapons Laboratory; this telescope represented the first successful implementation of closed-loop feedback control [2]. In 1993, a collaboration including the University of Arizona and the Osservatorio Astrofisico di Arcetri built the Large Binocular Telescope. The telescope comprises two sub-apertures, which each have an aperture of 8.4 m [3]. In 2004, three 0.21-m sub-apertures were used to construct the Adaptive Reconnaissance Golay-3 Optical Satellite, which was the product of an experiment carried out by the Massachusetts Institute of Technology [4]. In 2006, Lockheed–Martin Advanced Technology Center created the Star-9 system, which consists of nine 0.125-m sub-apertures [5]. In 2016, the Shanghai Astronomical Observatory of the CAS used the pyramid wave-front sensor to measure the piston error in the SAT [6]. In 2019, this group applied speckle imaging technology to the SAT, resulting in high-resolution reconstructed images [7]. In 2017, the Institute of Optics and Electronics of the Chinese Academy of Sciences (CAS) created a three sub-aperture synthetic imaging telescope based on phase detection; in this system, each sub-aperture is 127 mm [8,9]. In 2020, the segmented large-scale of lightweight diffractive telescope, whose primary mirror is made up of eight sub-mirrors and has a 352-mm aperture, was constructed by the same Institute [10]. In 2018, the National Astronomical Observatories of the CAS planned and constructed the Fizeau Imaging Interferometer testbed, which comprises three 100-mm sub-apertures [11,12]. In 2022, the Suzhou University of Science and Technology created a three sub-aperture telescope in which each sub-aperture is 80 mm [13]. Considerable research has also been devoted to phase correction techniques [14,15].

Although some organizations have built SATs, most of these telescopes lack the capability to track moving targets. Gimbals and fast steering mirrors (FSMs) are typically used in traditional telescope-based tracking systems. The field of view (FOV) of a SAT with an FSM installed without a gimbal is constrained, and the overall size of the system would increase considerably if a SAT was installed on a gimbal. Fortunately, the rotational double prism system (RDPS) enables a target to be tracked by adjusting the boresight via the rotation of the Risley prism; this system also benefits from having a compact construction and a wide FOV [16,17,18]. 

The RDPS research includes scanning [19,20,21,22], pointing [23,24,25,26], tracking [27,28,29,30,31,32], and similar applications. Calculating the rotation angle of each prism according to the azimuth and elevation angles is called the inverse solution. High-precision pointing and tracking are based on the inverse solution. The current inverse solution methods mainly include the analytical method [20,28,33,34], the numerical iteration method [25,35,36], and the numerical fitting method [37,38]. The two-step method is used in the majority of these [34]. In the first step of the two-step method, the rotation angle difference of the two prisms is found from the elevation angle of the beam deflection. In the second step, the synchronous rotation is calculated according to the angle difference obtained in the first step. The main difference among different inverse solution methods lies in the calculation of the first step. The numerical iterative method [30] is used for the inverse solution because the RDPS built in this paper does not involve the tracking of fast targets.

Based on our previous work, we recently built a set FDISAT platform, which is composed of three sub-apertures, and each sub-aperture is installed with a set of RDPSs. This study presents the results of pointing and tracking experiments performed on the FDISAT platform, which are not achievable with the majority of the existing SAT platforms. To the best of our knowledge, this is the first time that a RDPS has been used in a SAT to track a moving target. The present study is organized as follows. In Section 2, the FDISAT is introduced, and the error identification method is proposed. In Section 3, pointing and tracking experiments are introduced. The final section in this paper draws conclusions.

## 2. Fizeau Directly-Imaging Sparse-Aperture Telescope and Parameter Error Calibration

**Figure 1:**
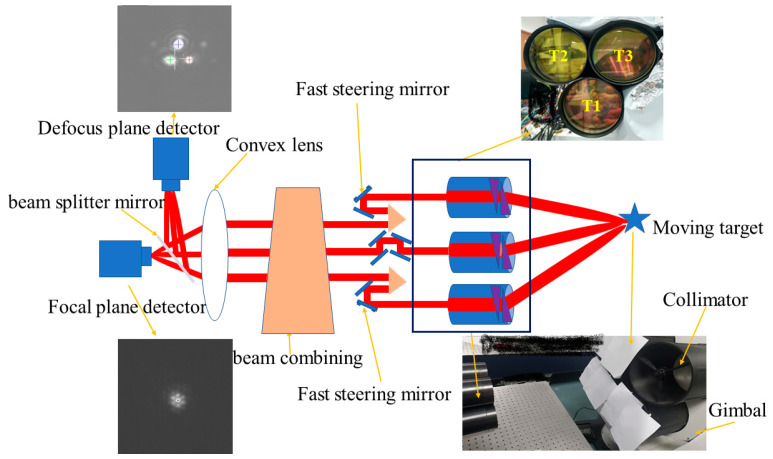
The Fizeau directly-imaging sparse aperture telescope platform.

Two elements can be observed in Figure 1: a gimbal and a FDISAT. The gimbal can emit a collimated beam through a collimator and can be rotated to simulate a moving target. The FDISAT receives the accurate position of target from the gimbal and undertakes pointing and tracking based on this position. The beam from the gimbal is first refracted by the RDPS installed in each sub-aperture; the beam is then reflected by the mirrors, and it is then passed through the beam combining system and the beam splitter. Finally, one part of the beam is captured by the detector at the focal plane, and another part of the beam is captured by the detector at the defocus plane. In this work, we only used the sub-apertures T1, T2, and T3 (see Figure 1) due to the limitations of the collimator apertures.

**Figure 2:**
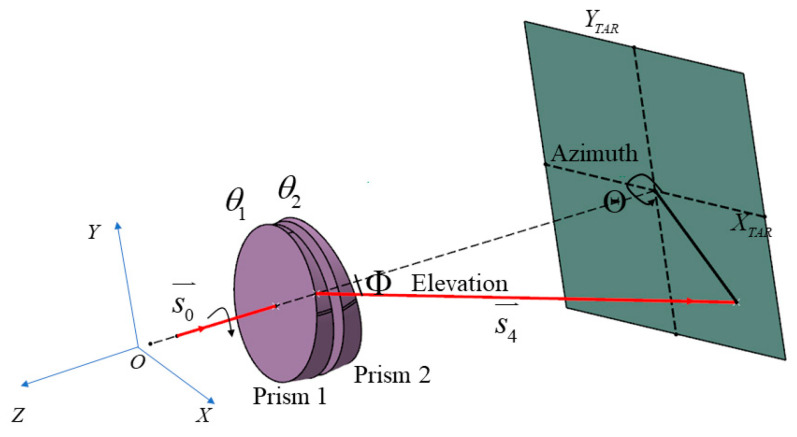
Beam deflection by a rotational double prism system.

Parametric errors in the RDPS will influence the pointing and tracking accuracy of the FDISAT. To identify these errors, we construct a mathematical model that includes the necessary error terms. Firstly, the coordinate transformation of the gimbal (only the azimuth axis is rotatable) relative to the RDPS can be expressed as:

(1) $$\Phi =\left|A_{G_{i}}-A_{G_{0}}\right|,$$

(2) $$\Theta =\left\{ \begin{array}{l} \pi ,\;A_{G_{i}}-A_{G_{0}}>0\\ 0,\;A_{G_{i}}-A_{G_{0}}\leq 0 \end{array}\right.,$$

where, Φ and Θ are the elevation and azimuth angles of the gimbal (target) in the coordinate system of the RDPS, respectively. $A_{G_{i}}$ is the current azimuth angle of the gimbal. $A_{G_{0}}$ is the azimuth angle of the gimbal when the light emitted by the collimator is vertically incident on the RDPS. Figure 2 shows a schematic of beam deflection by a RDPS. The azimuth and elevation angles of the beam deflection of the RDPS can be obtained by Snell’s law in vector form.

(3) $$\vec{s_{k}}=\frac{n_{k-1}}{n_{k}}[\vec{s_{k-1}}-(\vec{s_{k-1}}\cdot \vec{N_{k}})\cdot \vec{N_{k}}]-\vec{N_{k}}\sqrt{1-{\left(\frac{n_{k-1}}{n_{k}}\right)}^{2}[1-{\left(\vec{s_{k-1}}\cdot \vec{N_{k}}\right)}^{2}]},$$

where $\vec{s_{k}}$ and $\vec{s_{k-1}}$ represent the direction cosine of the *k*-th (*k* = 1, 2, …, 4) refracted emerging and incident beams, respectively. $n_{k-1}$ and $n_{k}$ represent the refractive indexes of the incident and exit mediums at the *k*-th refraction, respectively. ${\vec{N}}_{k}$ represents the normal vector of the prism surface at the *k*-th refraction, as shown in Equation (4).

(4) $$\begin{array}{l} \vec{N_{1}}=(\sin\alpha_{1}\cos\theta_{1},\sin\alpha_{1}\sin\theta_{1},\cos\alpha_{1})\\ \vec{N_{2}}=\vec{N_{3}}=(0,0,1)\\ \vec{N_{4}}=(-\sin\alpha_{1}\cos\theta_{2},-\sin\alpha_{2}\sin\theta_{2},\cos\alpha_{2}) \end{array},$$

where $\alpha_{1}$ and $\alpha_{2}$ are the wedge angles of prisms 1 and 2, respectively; $\theta_{1}$ and $\theta_{2}$ are the rotation angles of prisms 1 and 2, respectively. The emerging beam is identified as $\vec{s_{4}}=(K,L,M)$ when the incident beam $\vec{s_{0}}=(0,0,-1)$ is refracted four times. The azimuth angle Θ and elevation angle Φ of the RDPS for the beam deflection can be expressed as,

(5) $$\Theta =\left\{ \begin{array}{l} arc\tan(\frac{L}{K}),K\geq 0\\ arc\tan(\frac{L}{K})+\pi ,K<0 \end{array}\right..$$

(6) Φ=arccos(−M).

Here, for clarity, the azimuth angle, Θ, and elevation angle, Φ, are assumed to be obtained via the use of Equations (7) and (8), respectively. The details of the calculation of these quantities can be found in Appendix A.

(7) $$\Theta =f_{A}(\theta_{1},\theta_{2},\alpha_{1},\alpha_{1},n_{1},n_{2}),$$

(8) $$\Phi =f_{E}(\theta_{1},\theta_{2},\alpha_{1},\alpha_{1},n_{1},n_{2}).$$

The following error sources are considered in the RDPS: the difference between the zero positions of the two prisms and the zero positions of the encoders, which are referred to as $\Delta \theta_{1}$ and $\Delta \theta_{2}$, respectively; the difference between the theoretical and actual values of the wedge angles of the two prisms, which are referred to here as $\Delta \alpha_{1}$ and $\Delta \alpha_{2}$, respectively; and the difference between the theoretical and actual values of the refractive index of the two prisms, which are referred to as $\Delta n_{1}$ and $\Delta n_{2}$, respectively. Some parameters in Equations (3) and (4) can be expressed as:

(9) $$\left\{ \begin{array}{l} \theta_{1}=\theta_{10}+\Delta \theta_{1}\\ \theta_{2}=\theta_{20}+\Delta \theta_{2}\\ \alpha_{1}=\alpha_{10}+\Delta \alpha_{1}\\ \alpha_{2}=\alpha_{20}+\Delta \alpha_{2}\\ n_{1}=n_{10}+\Delta n_{1}\\ n_{2}=n_{20}+\Delta n_{2} \end{array}\right.,$$

where $\theta_{10}$, $\alpha_{10}$, and $n_{10}$ are the theoretical values of the zero position, wedge angle, and refractive index of prism 1, respectively; $\theta_{20}$, $\alpha_{20}$, and $n_{20}$ are the theoretical values of the zero position, wedge angle, and refractive index of prism 2, respectively. For the RDPS used in this paper, $\theta_{10}=\theta_{20}=0$, $\alpha_{10}=\alpha_{20}=6^{\circ }$, and $n_{10}=n_{20}=1.45012$.

**Figure 3:**
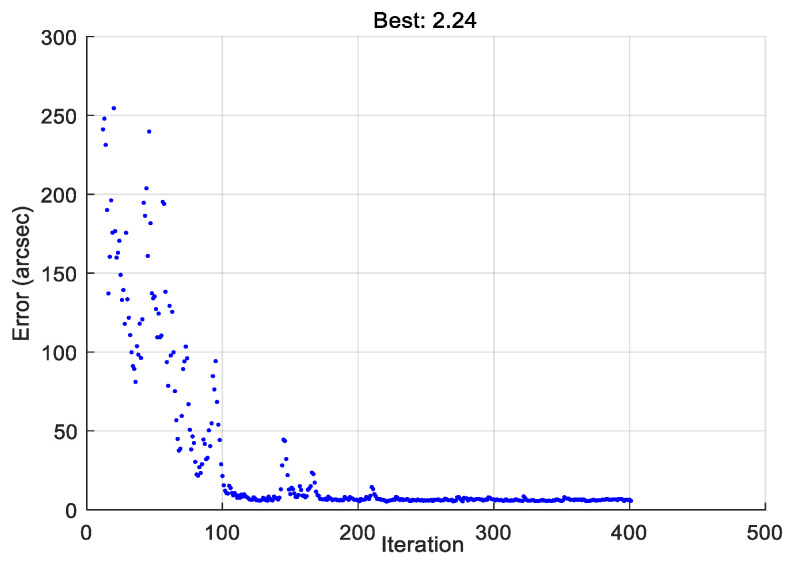
Curve of error variation during optimization.

By rotating the gimbal to various positions (which simulates the target in various positions), we can precisely identify the errors. The azimuth,$\Theta_{g\_i}$, and elevation, $\Phi_{g\_i}$, of the gimbal feedback relative to the RDPS and the encoder values, $\theta_{1}{}_{i}$ and $\theta_{2}{}_{i}$, for prisms 1 and 2, respectively, of the RDPS, were then recorded. The optimization criterion that was employed is given by,

(10) $$\begin{array}{l} \min\sum_{i=1}^{n}\{{\left[f_{A}(\theta_{1}{}_{i},\Delta \theta_{1},\theta_{2i},\Delta \theta_{2},\Delta \alpha_{1},\Delta \alpha_{2},\Delta n_{1},\Delta n_{2})-\Theta_{g\_i}\right]}^{2}\\ \;+[f_{E}(\theta_{1}{}_{i},\Delta \theta_{1},\theta_{2i},\Delta \theta_{2},\Delta \alpha_{1},\Delta \alpha_{2},\Delta n_{1},\Delta n_{2})-\Phi_{g\_i}{]}^{2}\} \end{array},$$

where *n* is the total number of sampling positions. Taking T1 as an example, a total of 31 groups of data were collected from various positions. Figure 3 displays the variation of error when a genetic algorithm is used to optimize Equation (10). As the number of iterations increases, the error converges to near the minimum value (see in Figure 3). The minimum value of Equation (10) in the whole optimization process is 2.24 arcsec. The set of variables that minimizes the value of Equation (10) is chosen as the parameter error.

The results of our investigation of prism parameter errors are presented in Table 1. According to Table 1, there are considerable errors (>50 deg) between the zero positions of the prism and the encoder for each sub-aperture. As a result, it takes a long time for us to capture the target during the identification process. The wedge angle of the prism has a milliradian error. If these errors are not calibrated, it is difficult to produce high-precision pointing and tracking results in the following work.

## 3. Pointing and Tracking Experiment

### 3.1. Pointing Experiment

**Figure 4:**
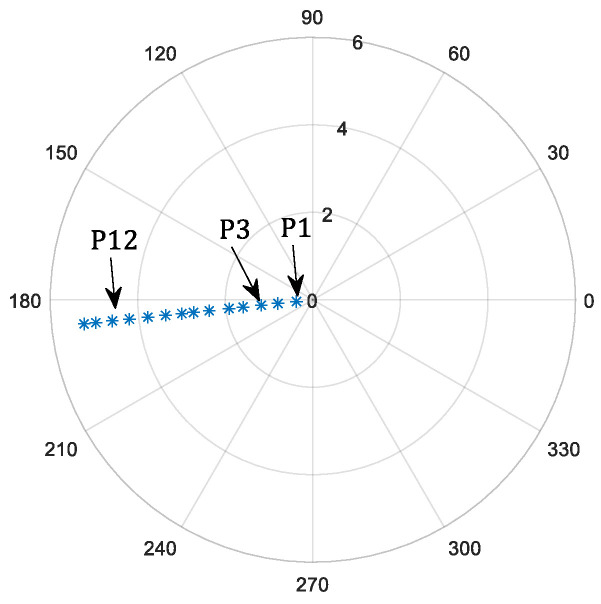
Position of the target in polar coordinate system, from right to left, named P1, P2, …, P14 respectively.

High-precision pointing can reduce the time required to capture the target. Therefore, one of the key metrics considered here in evaluating the FDISAT is its pointing precision. During the pointing experiment, the gimbal is first rotated to a given position, and then, according to the position selected, the rotational angle required by the RDPS is calculated using the numerical iteration method [30]. The spot offset measured by the defocus plane detector was then recorded. The gimbal was then rotated to the next position, and the measurement process was repeated. The RMSE of the pointing in the x-direction, $Potx_{RMSE}$, y-direction, $Poty_{RMSE}$, and total, $Potr_{RMSE}$, are defined as follows,

(11) $$Potx_{RMSE}=\sqrt{\frac{1}{N}\sum_{i=1}^{N}{\left(ccdx_{i}-ccdx_{0}\right)}^{2}}\times equ_{x},$$

(12) $$Poty_{RMSE}=\sqrt{\frac{1}{N}\sum_{i=1}^{N}{\left(ccdy_{i}-ccdy_{0}\right)}^{2}}\times equ_{y},$$

(13) $$Potr_{RMSE}=\sqrt{Potx_{RMSE}{}^{2}+Poty_{RMSE}{}^{2}},$$

where *N* is the total number of sampling position; $ccdx_{i}$ and $ccdy_{i}$ represent the spot offset in the x- and y-directions, respectively, as measured by the defocus plane detector at the i-th sampling position; $ccdx_{0}$ and $ccdy_{0}$ represent the x- and y-direction values, respectively, of each sub-aperture at the center of the defocus plane detector; and $equ_{x}$ and $equ_{y}$ represent the equivalent information in the x- and y-directions, respectively, and their values are both 0.40 arcsec. As indicated in Figure 4, a total of 14 sampling positions were selected for use in the pointing experiment. The sampling positions were distributed along a tilted line because the gimbal can only be controlled precisely in a one-dimensional space, and there is a specific tilt angle between the FDISAT and the gimbal. The quality of the spot received by the sub-aperture is weak in the region near the azimuth angle of 0°, so the target position in this region was not selected for verification. Table 2 shows the RMSE of pointing measurements. As can be seen from Table 2, the maximum total RMSE of pointing was found to be 8.22 arcsec.

**Figure 5:**
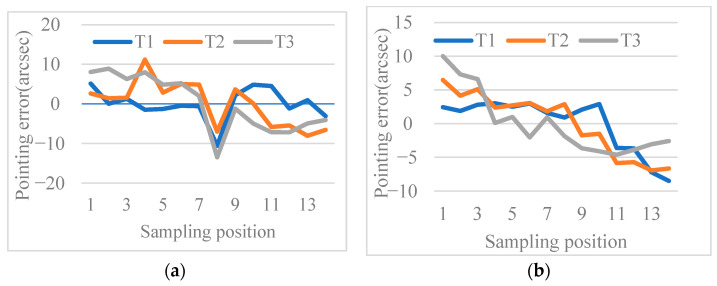
Pointing error of each sampling position for each sub-aperture: (**a**) x-direction and (**b**) y-direction.

**Figure 6:**
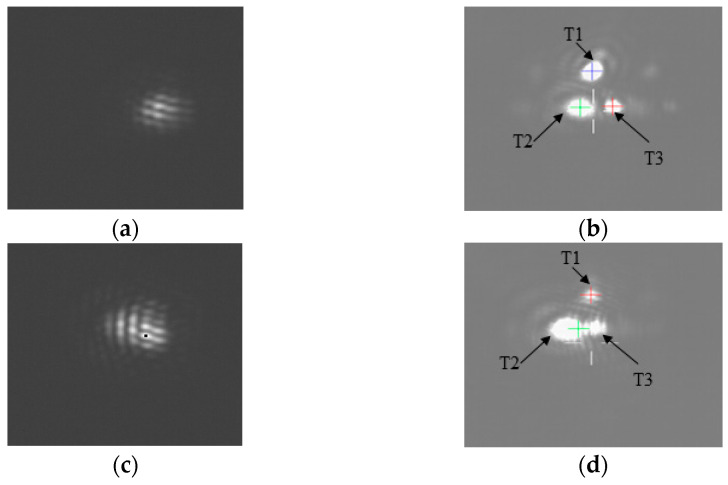
Imaging results obtained from the focal plane detector and the defocus plane detector at different pointing positions; the images were taken from the position of P3 at the (**a**) focal plane and (**b**) defocus plane and the position of P12 at the (**c**) focal plane and (**d**) defocus plane.

Figure 5 shows the pointing error as a function of position. The maximum pointing error is less than 15 arcsec for all sampling positions. The focal plane measurements (Figure 6a,c), show that after the target is captured via pointing, some interference fringes are present on the focal plane. This shows that the pointing accuracy of the FDISAT meets the application requirements. It can be seen in Figure 6d that some spots on the defocus plane are too close to each other; this leads to errors in the centroid extraction and increased the pointing error. In the case of a single spot in the detector, the RMSEs of pointing in the x- and y-directions of T2 are only 1.35 and 1.36 arcsec, respectively. The pointing results demonstrate that even if the target is in a different position, our SAT system can adjust the boresight by rotating the prism for the purpose of observing the target.

### 3.2. Tracking Experiment

**Figure 7:**
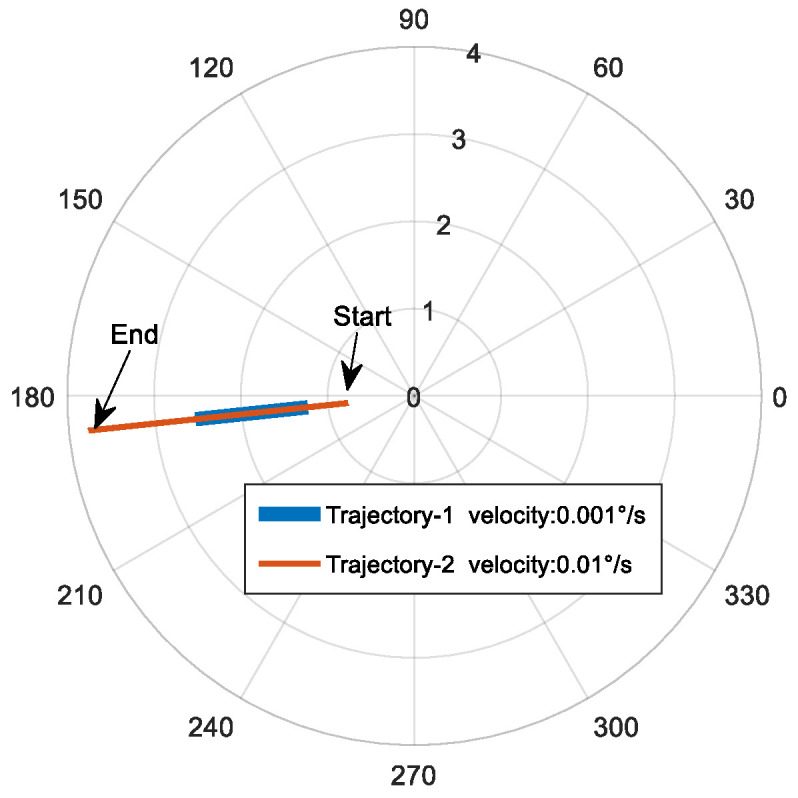
The trajectory of the target depicted in a polar coordinate system (the position of the target moves from right to left).

In the tracking experiment, it is first necessary to set a trajectory for the gimbal, which serves as a guidance position for the FDISAT. Once the target enters the FOV of the detector, each sub-aperture can then perform closed-loop tracking based on the spot offset obtained from the defocus detector. Figure 7 shows the two chosen trajectories for the tracking experiments undertaken here; these trajectories are referred to here as trajectory-1 and trajectory-2. Trajectory-1 and trajectory-2 have a velocity of $0.001^{\circ }/$s and $0.01^{\circ }/$s, respectively.

**Figure 8:**
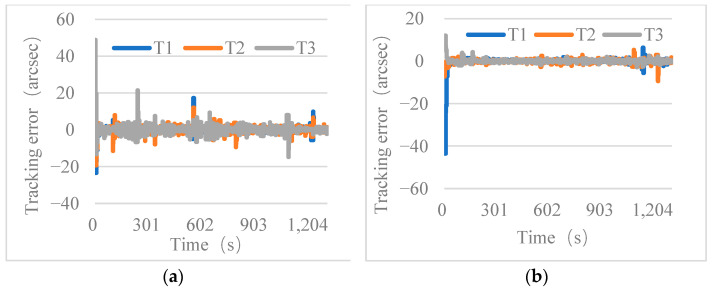

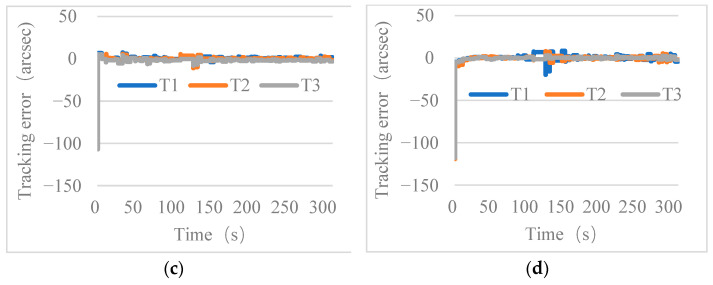
The tracking error as a function of time for each sub-aperture for (**a**) x-direction of trajectory-1, (**b**) y-direction of trajectory-1, (**c**) x-direction of trajectory-2, (**d**) y-direction of trajectory-2.

The tracking errors in the x- and y-directions for each sub-aperture are shown in Figure 8. The maximum tracking error for the FDISAT during the closed-loop tracking of a moving target was less than 25 arcsec. The tracking error of the FDISAT remains ~2 arcsec during the target movement. Equations (14)–(16) show the calculation of the RMSE of tracking for each sub-aperture using the tracking error data after 20 s of closed-loop tracking.

(14) $$Trkx_{RMSE}=\sqrt{\frac{1}{N}\sum_{i=1}^{N}{\left(ccdx_{i}-ccdx_{0}\right)}^{2}}\times equ_{x},$$

(15) $$Trky_{RMSE}=\sqrt{\frac{1}{N}\sum_{i=1}^{N}{\left(ccdy_{i}-ccdy_{0}\right)}^{2}}\times equ_{y},$$

(16) $$Trkr_{RMSE}=\sqrt{Trkx_{RMSE}{}^{2}+Trky_{RMSE}{}^{2}},$$

where $Trkx_{RMSE}$, $Trky_{RMSE}$, and $Trkr_{RMSE}$ represent the RMSE of tracking in the *x*-direction and *y*-direction, and total, respectively. Table 3 shows the values of the RMSE of tracking. From Table 3, it can be seen that the maximum values of the RMSE of tracking for those sub-apertures is 4.23 arcsec.

**Figure 9:**
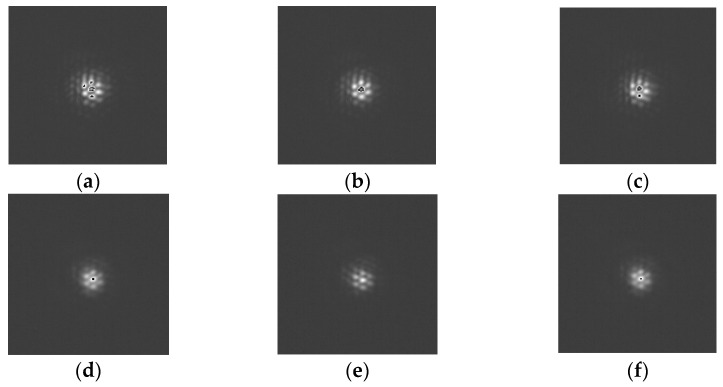
Images obtained from the focal plane detector feedback during the tracking of trajectory-1. The images correspond to a tracking time, t, of (**a**) 180 s, (**b**) 420 s, (**c**) 540 s, (**d**) 780 s, (**e**) 900 s, and (**f**) 1260 s.

**Figure 10:**
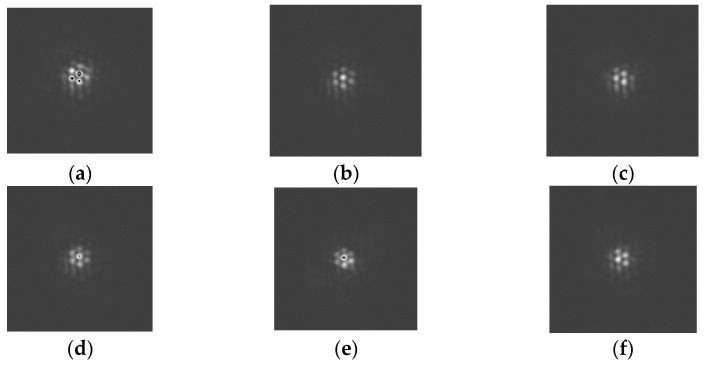
Images obtained from the focal plane detector feedback during the tracking of trajectory-2. The images correspond to a tracking time, t, of (**a**) 50 s, (**b**) 100 s, (**c**) 150 s, (**d**) 200 s, (**e**) 250 s, and (**f**) 300 s.

Figure 9 and Figure 10 show the imaging results from the focal plane from the tracking of trajectory-1 and trajectory-2. Figure 9 and Figure 10 indicate that the focus plane was in a state of interference for the majority of the tracking experiment, resulting in more obvious interference fringes. The interference fringe in Figure 10 is a little worse than that in Figure 9. This can be explained by noting that the tracking performance of the system is reduced by the increased velocity of the target. Due to the unstable nature of the light source (produced by the laser), the detector detects different light intensities at different positions. The interference fringes on the focal plane demonstrate the effectiveness of the FDISAT in tracking a moving target.

The FDISAT system, after error calibration, obtained a pointing error of ~ 5 arcsec in a consistent pointing experiment. When the target was in a different position, partial interference fringes appear on the focus detector by adjusting the boresight of the RDPS. In the tracking experiments, targets with different motion velocities were tracked for long periods of time, and a tracking error of ~2 arcsec was achieved. Interference fringes were also noticeable on the focal plane during the moving of the target. The experimental results for pointing and tracking demonstrate the effectiveness of the FDISAT in observing a moving target.

## 4. Conclusions

This paper introduced a FDISAT comprised of three sub-apertures. A set of RDPSs were installed in each sub-aperture to enable pointing and tracking of moving targets. To evaluate the pointing and tracking precision of the system, three sub-apertures were selected, and the parameter error of the prisms were calibrated. The maximum RMSE for pointing was found to be 8.22 arcsec, and the maximum RMSE for tracking a moving target was found to be 4.23 arcsec. The focal plane detector was able to capture obvious interference fringes as the target moved, which demonstrates the effectiveness of our technique. The results of our experiment offer a method for SAT observation of moving targets. Future work will focus on the secondary correction of errors using FSM and inertial sensors to further improve the tracking accuracy of the system.

## Figures and Tables

**Table 1:** Parameter errors for each sub-aperture.

Error Term	T1	T2	T3
$\Delta \theta_{1}$ (deg)	270.83	55.12	336.10
$\Delta \theta_{2}$ (deg)	332.24	107.96	307.04
$\Delta \alpha_{1}$ (mrad)	−0.18	−0.67	−0.95
$\Delta \alpha_{2}$ (mrad)	−2.02	2.74	2.69
$\Delta n_{1}$	0.01	0.01	−0.01
$\Delta n_{2}$	0.02	−0.01	−0.01

**Table 2:** RMSE of pointing of each sub-aperture.

Apertures	$Poty_{RMSE}\;(\mathrm{arcsec})$	$Poty_{RMSE}\;(\mathrm{arcsec})$	$Potr_{RMSE}\;(\mathrm{arcsec})$
T1	3.83	3.86	5.44
T2	5.53	4.51	7.14
T3	6.84	4.55	8.22

**Table 3:** RMSE of tracking for each sub-aperture.

Velocity (°/s)	Aperture	$Trkx_{RMSE}\;(\mathrm{arcsec})$	$Trky_{RMSE}\;(\mathrm{arcsec})$	$Trkr_{RMSE}\;(\mathrm{arcsec})$
0.001	T1	1.51	0.79	1.70
	T2	1.98	0.94	2.19
	T3	2.35	0.76	2.47
0.01	T1	2.06	3.69	4.23
	T2	2.10	2.07	2.95
	T3	2.38	1.07	2.61

## Data Availability

Data underlying the results presented in this paper are not publicly available at this time but may be obtained from the authors upon reasonable request.

## Appendix A

For the first refraction, the incident medium is air, and the exit medium is prism1. When k = 1, Equation (3) can be expressed as:

(A1) $$\vec{s_{1}}=\frac{n_{0}}{n_{1}}[\vec{s_{0}}-(\vec{s_{0}}\cdot \vec{N_{1}})\vec{N_{1}}]-\vec{N_{1}}\sqrt{1-{\left(\frac{n_{0}}{n_{1}}\right)}^{2}[1-{\left(\vec{s_{0}}\cdot \vec{N_{1}}\right)}^{2}]},$$

where $n_{0}=1$, $\vec{s_{0}}={\left(0,0,-1\right)}^{T}$, $\vec{N_{1}}=(\sin\alpha_{1}\cos\theta_{1},\sin\alpha_{1}\sin\theta_{1},\cos\alpha_{1})$. Equation (A1) can be expressed as:

(A2) $$\begin{array}{l} \vec{s_{1}}=\frac{1}{n_{1}}[\left(\begin{array}{l} 0\\ 0\\ -1 \end{array}\right)-(\left(\begin{array}{l} 0\\ 0\\ -1 \end{array}\right)\cdot \left(\begin{array}{l} \sin\alpha_{1}\cos\theta_{1}\\ \sin\alpha_{1}\sin\theta_{1}\\ \cos\alpha_{1} \end{array}\right))\left(\begin{array}{l} \sin\alpha_{1}\cos\theta_{1}\\ \sin\alpha_{1}\sin\theta_{1}\\ \cos\alpha_{1} \end{array}\right)]\\ \;-\left(\begin{array}{l} \sin\alpha_{1}\cos\theta_{1}\\ \sin\alpha_{1}\sin\theta_{1}\\ \cos\alpha_{1} \end{array}\right)\sqrt{1-{\left(\frac{1}{n_{1}}\right)}^{2}(1-\cos^{2}\alpha_{1})} \end{array}$$

Let $c_{1}=\frac{1}{n_{r2}}\cos\alpha_{1}-\sqrt{1-{\left(\frac{1}{n_{r2}}\right)}^{2}(1-\cos^{2}\alpha_{1})}$, then, Equation (A2) can be expressed as:

(A3) $$\vec{s_{1}}==\left(\begin{array}{l} c_{1}\sin\alpha_{1}\cos\theta_{1}\\ c_{1}\sin\alpha_{1}\sin\theta_{1}\\ -\frac{1}{n_{r2}}+c_{1}\cos\alpha_{1} \end{array}\right).$$

$\vec{s_{2}},\vec{s_{3}},\vec{s_{4}}$ can also be calculated from Equations (A1)–(A3).
